# Development and experimental test of support vector machines virtual screening method for searching Src inhibitors from large compound libraries

**DOI:** 10.1186/1752-153X-6-139

**Published:** 2012-11-23

**Authors:** Bucong Han, Xiaohua Ma, Ruiying Zhao, Jingxian Zhang, Xiaona Wei, Xianghui Liu, Xin Liu, Cunlong Zhang, Chunyan Tan, Yuyang Jiang, Yuzong Chen

**Affiliations:** 1 The Key Laboratory of Chemical Biology, Guangdong Province, The Graduate School at Shenzhen, Tsinghua University, Shenzhen, Guangdong, 518055, People’s Republic of China; 2 Computation and Systems Biology, Singapore-MIT Alliance, National University of Singapore, E4-04-10, 4 Engineering Drive 3, Singapore, 117576, Singapore; 3 Bioinformatics and Drug Design Group, Department of Pharmacy, Centre for Computational Science and Engineering, National University of Singapore, Blk S16, Level 8, 3 Science Drive 2, Singapore, 117543, Singapore; 4 Central Research Institute of China Chemical Science and Technology, 20 Xueyuan Road, Haidian District, Beijing, 100083, People’s Republic of China

**Keywords:** Src, c-src, Computer aided drug design, Kinase inhibitor, Virtual screening, Support vector machine

## Abstract

**Background:**

Src plays various roles in tumour progression, invasion, metastasis, angiogenesis and survival. It is one of the multiple targets of multi-target kinase inhibitors in clinical uses and trials for the treatment of leukemia and other cancers. These successes and appearances of drug resistance in some patients have raised significant interest and efforts in discovering new Src inhibitors. Various *in-silico* methods have been used in some of these efforts. It is desirable to explore additional *in-silico* methods, particularly those capable of searching large compound libraries at high yields and reduced false-hit rates.

**Results:**

We evaluated support vector machines (SVM) as virtual screening tools for searching Src inhibitors from large compound libraries. SVM trained and tested by 1,703 inhibitors and 63,318 putative non-inhibitors correctly identified 93.53%~ 95.01% inhibitors and 99.81%~ 99.90% non-inhibitors in 5-fold cross validation studies. SVM trained by 1,703 inhibitors reported before 2011 and 63,318 putative non-inhibitors correctly identified 70.45% of the 44 inhibitors reported since 2011, and predicted as inhibitors 44,843 (0.33%) of 13.56M PubChem, 1,496 (0.89%) of 168 K MDDR, and 719 (7.73%) of 9,305 MDDR compounds similar to the known inhibitors.

**Conclusions:**

SVM showed comparable yield and reduced false hit rates in searching large compound libraries compared to the similarity-based and other machine-learning VS methods developed from the same set of training compounds and molecular descriptors. We tested three virtual hits of the same novel scaffold from in-house chemical libraries not reported as Src inhibitor, one of which showed moderate activity. SVM may be potentially explored for searching Src inhibitors from large compound libraries at low false-hit rates.

## Background

Src promotes tumour invasion and metastasis, facilitates VEGF-mediated angiogenesis and survival in endothelial cells, and enhances growth factor driven proliferation in fibroblasts [1]. It is one of the multiple kinase targets of a number of multi-target kinase inhibitors effective in the clinical treatment of leukemia and in clinical trials of other cancers [2-4]. The successes and problems of these inhibitors have raised significant interest and efforts in discovering new Src inhibitors [5-7]. Several *in-silico* methods have been used for facilitating the search and design of Src inhibitors, which include pharmacophore [8], Quantitative Structure Activity Relationship (QSAR) [9], and molecular docking [6].

While these *in-silico* methods have shown impressive capability in the identification of potential Src inhibitors, their applications may be affected by such problems as the vastness and sparse nature of chemical space needing to be searched, complexity and flexibility of target structures, difficulties in accurately estimating binding affinity and solvation effects on molecular binding, and limited representativeness of training active compounds [10-12]. It is desirable to explore other *in-silico* methods that complement these methods by expanded coverage of chemical space, increased screening speed, and reduced false-hit rates without necessarily relying on the modelling of target structural flexibility, binding affinity and salvation effects.

Support vector machines (SVM) has recently been explored as a promising ligand-based virtual screening (VS) method that produces high yields and low false-hit rates in searching active agents of single and multiple mechanisms from large compound libraries [13] and in identifying active agents of diverse structures [13-17]. Good VS performance can also be achieved by SVM trained from sparsely distributed active compounds [18]. SVM classifies active compounds based on the separation of active and inactive compounds in a hyperspace constructed by their physicochemical properties rather than structural similarity to active compounds *per se*, which has the advantage of not relying on the accurate computation of structural flexibility, activity-related features, binding affinity and solvation effects. Moreover, the fast speed of SVM enables efficient search of vast chemical space. Therefore, SVM may be a potentially useful VS tool to complement other *in-silico* methods for searching Src inhibitors from large libraries.

In this work, we developed a SVM VS model for identifying Src inhibitors, and evaluated its performance by both 5-fold cross validation test and large compound database screening test. In 5-fold cross validation test, a dataset of Src inhibitors and non-inhibitors was randomly divided into 5 groups of approximately equal size, with 4 groups used for training a SVM VS tool and 1 group used for testing it, and the test process is repeated for all 5 possible compositions to derive an average VS performance. In large database screening test, a SVM VS tool was developed by using Src inhibitors published before 2011, its yield (percent of known inhibitors identified as virtual-hits) was estimated by using Src inhibitors reported since 2011 and not included in the training datasets, virtual-hit rate and false-hit rate in searching large libraries were evaluated by using 13.56M PubChem and 168K MDDR compounds, and an additional set of 9,305 MDDR compounds similar in structural and physicochemical properties to the known Src inhibitors.

Moreover, VS performance of SVM was compared to those of two similarity-based VS methods, Tanimoto similarity searching and k nearest neighbour (kNN), and an alternative but equally popularly used machine learning method, probabilistic neural network (PNN) method, based on the same training and testing datasets (same sets of PubChem and MDDR compounds) and molecular descriptors. In a study that compares the performance of SVM to 16 classification methods and 9 regression methods, it has been reported that SVMs shows mostly good performances both on classification and regression tasks, but other methods proved to be very competitive [19]. Therefore, it is useful to evaluate the VS performance of SVM in searching large compound libraries by comparison with those of both similarity-based approaches and other typical machine learning method.

PubChem and MDDR contain high percentages of inactive compounds significantly different from the known Src inhibitors, and the easily distinguishable features may make VS enrichments artificially good [20]. Therefore, VS performance may be more strictly tested by using subsets of compounds that resemble the physicochemical properties of the known Src inhibitors so that enrichment is not simply a separation of trivial physicochemical features [21]. To further evaluate whether our SVM VS tool predict Src inhibitors and non-inhibitors rather than membership of certain compound families, distribution of the predicted active and inactive compounds in the compound families were analyzed.

## Materials and methods

### Compound collections and construction of training and testing datasets

**Figure 1:**
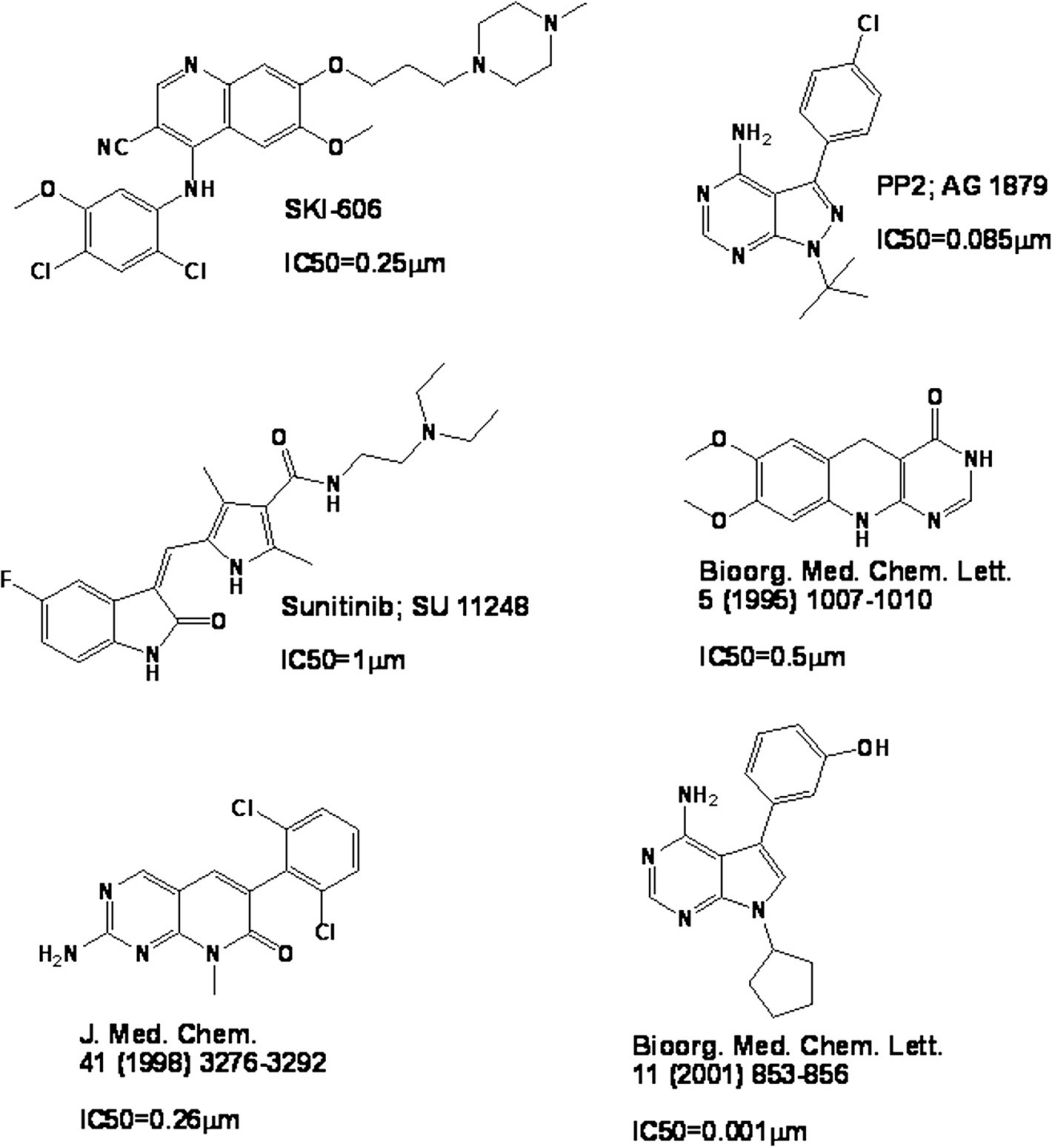
The structures of representative c-Src inhibitors.

We collected 1,703 Src inhibitors reported before 2011, with IC50<10 μM, from the literatures [22-26] and the BindingDB database [27]. The inhibitor selection criterion of IC50<10 μM was used because it covers most of the reported HTS and VS hits [28,29]. The structures of representative Src inhibitors are shown in Figure 1. As few non-inhibitors have been reported, putative non-inhibitors were generated by using our method for generating putative inactive compounds [13,18]. This method requires no knowledge of known inactive compounds and active compounds of other target classes, which enables more expanded coverage of the “non-inhibitor” chemical space. Although the yet-to-be-discovered inhibitors are likely distributed in some of these “non-inhibitor” families, a substantial percentage of these inhibitors are expected to be identified as inhibitors rather than non-inhibitors even-though representatives of their families are putatively assigned as non-inhibitors [13]. 13.56M PubChem and 168 K MDDR compounds were grouped into 8,423 compound families by clustering them in the chemical space defined by their molecular descriptors [30,31]. The number of generated families is consistent with the 12,800 compound-occupying neurons (regions of topologically close structures) for 26.4 million compounds of up to 11 atoms [32], and the 2,851 clusters for 171,045 natural products [33].

Our collected Src inhibitors are distributed in 493 families. Because of the extensive efforts in searching kinase inhibitors from known compound libraries, the number of undiscovered Src inhibitor families in PubChem and MDDR databases is expected to be relatively small, most likely no more than several hundred families. The ratio of the discovered and undiscovered inhibitor families (hundreds) and the families that contain no known Src inhibitor (8,423 based on the current versions of PubChem and MDDR) is expected to be <15%. Therefore, putative non-inhibitor training dataset can be generated by extracting a few representative compounds from each of those families that contain no known inhibitor, with a maximum possible “wrong” classification rate of <15% even when all of the undiscovered inhibitors are misplaced into the non-inhibitor class. The noise level generated by up to 15% “wrong” negative family representation is expected to be substantially smaller than the maximum 50% false-negative noise level tolerated by SVM [16]. Based on earlier studies [13,18] and this work, it is expected that a substantial percentage of the un-discovered inhibitors in the putative “non-inhibitor” families can be classified as inhibitor despite their family representatives are placed into the non-inhibitor training sets.

In the database screening test, 60.1% of the families that contain Src inhibitors reported since 2011 [34-39] are not covered by the Src inhibitor training dataset (inhibitors reported before 2011). The representative compounds of these families, none of which happen to be Src inhibitor, were deliberately placed into the inactive training sets because the inhibitors in these families are not supposed to be known in our study. As shown in earlier studies [13,18] and in this work, a substantial percentage of the inhibitors in these misplaced inhibitor-containing “non-inhibitor” families were predicted as inhibitors by our SVM VS tool. Moreover, a small percentage of the compounds in these putative non-inhibitor datasets are expected to be un-reported and un-discovered inhibitors, their presence in these datasets is not expected to significantly affect the estimated false hit rate of SVM.

### Molecular descriptors

Molecular descriptors are quantitative representations of structural and physicochemical features of molecules, which have been extensively used in deriving structure-activity relationships [40,41], quantitative structure activity relationships [42,43] and VS tools [44-51]. A total of 98 1D and 2D descriptors derived by using our software [52] were used in this work. These descriptors and the relevant references are given in Table 1, which include 18 descriptors in the class of simple molecular properties, 3 descriptors in the class of chemical properties, 35 descriptors in the class of molecular connectivity and shape, 42 descriptors in the class of electro-topological state.

**Table 1:** Molecular descriptors used in this work

**Descriptor class**	**No of descriptors in class**	**Descriptors**
Simple molecular properties [53]	18	Number of C,N,O,P,S, Number of total atoms, Number of rings, Number of bonds, Number of non-H bonds, Molecular weight,, Number of rotatable bonds, number of H-bond donors, number of H-bond acceptors, Number of 5-member aromatic rings, Number of 6-member aromatic rings, Number of N heterocyclic rings, Number of O heterocyclic rings, Number of S heterocyclic rings.
Chemical properties [54]	3	Sanderson electronegativity, Molecular polarizability, aLogp
Molecular Connectivity and shape [53,55]	35	Schultz molecular topological index, Gutman molecular topological index, Wiener index, Harary index, Gravitational topological index, Molecular path count of length 1–6, Total path count, Balaban Index J, 0-2th valence connectivity index, 0-2th order delta chi index, Pogliani index, 0-2th Solvation connectivity index, 1-3th order Kier shape index, 1-3th order Kappa alpha shape index, Kier Molecular Flexibility Index, Topological radius, Graph-theoretical shape coefficient, Eccentricity, Centralization, Logp from connectivity.
Electro-topological state [53,56]	42	Sum of Estate of atom type sCH3, dCH2, ssCH2, dsCH, aaCH, sssCH, dssC, aasC, aaaC, sssC, sNH3, sNH2, ssNH2, dNH, ssNH, aaNH, dsN, aaN, sssN, ddsN, aOH, sOH, ssO, sSH; Sum of Estate of all heavy atoms, all C atoms, all hetero atoms, Sum of Estate of H-bond acceptors, Sum of H Estate of atom type HsOH, HdNH, HsSH, HsNH2, HssNH, HaaNH, HtCH, HdCH2, HdsCH, HaaCH, HCsats, HCsatu, Havin, Sum of H Estate of H-bond donors

### Support vector machines method

**Figure 2:**
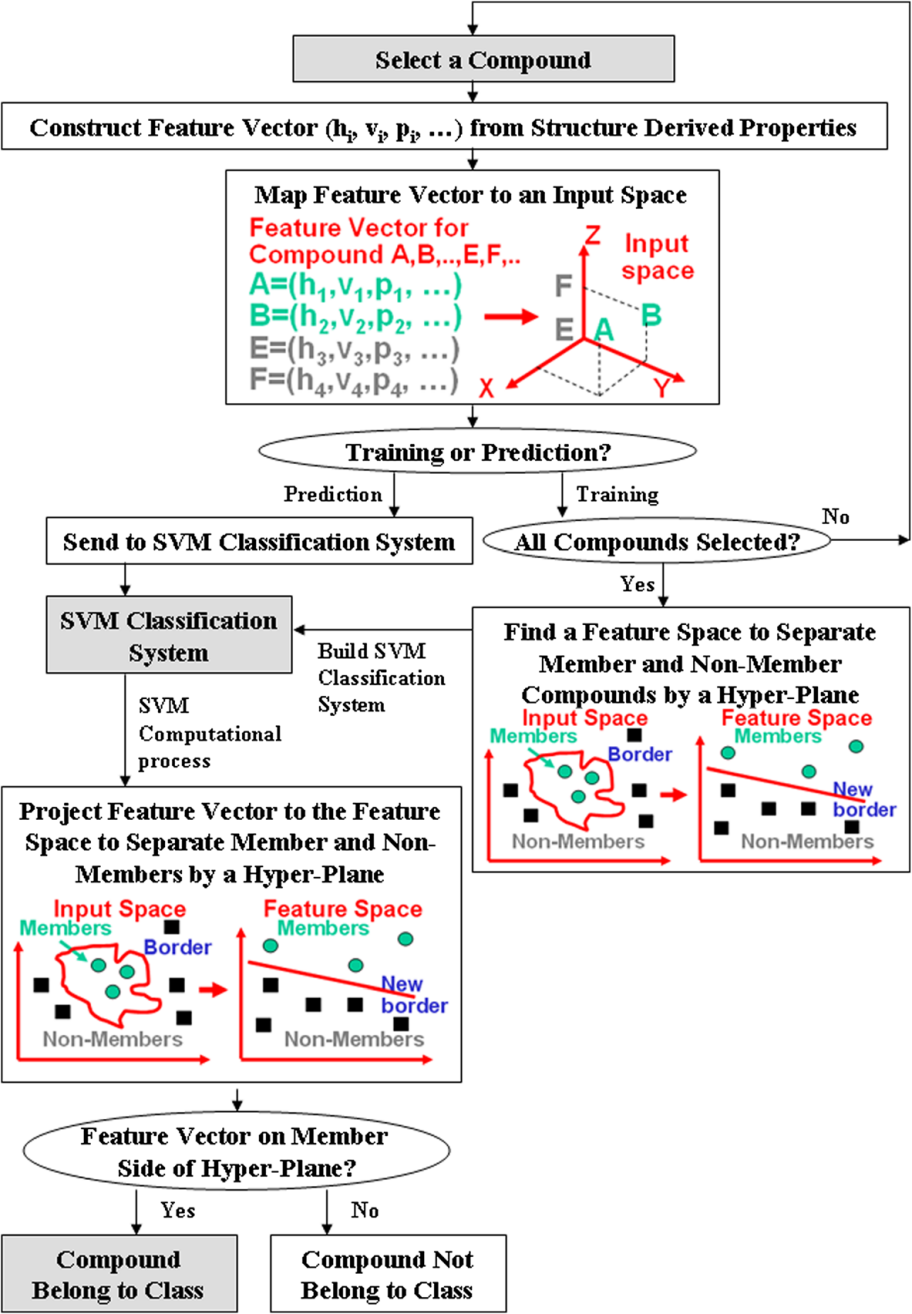
**The process of training and using a SVM VS model for screening compounds.** Schematic diagram is illustrating the process of the training a prediction model and using it for predicting active compounds of a compound class from their structurally-derived properties (molecular descriptors) by using support vector machines. A, B, E, F and (h_j_, p_j_, vj,…) represents such structural and physicochemical properties as hydrophobicity, volume, polarizability, etc.

The process of training and using a SVM VS model for screening compounds based on their molecular descriptors is schematically illustrated in Figure 2. SVM is based on the structural risk minimization principle of statistical learning theory [57,58], which consistently shows outstanding classification performance, is less penalized by sample redundancy, and has lower risk for over-fitting [59,60]. In linearly separable cases, SVM constructs a hyper-plane to separate active and inactive classes of compounds with a maximum margin. A compound is represented by a vector ***x***_*i*_ composed of its molecular descriptors. The hyper-plane is constructed by finding another vector **w** and a parameter *b* that minimizes ‖**w**‖^2^ and satisfies the following conditions:

(1) $$w\cdot x_{i}+b\geq +1,\text{for}\;y_{i}=+1\;\text{Class 1}\;\left(\text{active}\right)$$

(2) $$w\cdot x_{i}+b\leq -1,\text{for}\;y_{i}=-1\;\text{Class 2}\;\left(\text{inactive}\right)$$

where *y*_*i*_ is the class index, **w** is a vector normal to the hyperplane, |*b*|/‖**w**‖ is the perpendicular distance from the hyperplane to the origin and ‖**w**‖^2^ is the Euclidean norm of **w**. Base on **w** and *b*, a given vector ***x*** can be classified by *f*(*x*) = *sign*[(**w** · **x**) + *b*. A positive or negative *f(****x****)* value indicates that the vector **x** belongs to the active or inactive class respectively.

In nonlinearly separable cases, which almost always occur in classifying compounds of diverse structures [14-17,50,61-63], SVM maps the input vectors into a higher dimensional feature space by using a kernel function K(**x**_*i*_, **x**_*j*_). We used RBF kernel $K\left(x_{i},x_{j}\right)=e^{-{\left\|x_{j}-x_{i}\right\|}^{2}/2\sigma^{2}}$ which has been extensively used and consistently shown better performance than other kernel functions [64-66]. Linear SVM can then applied to this feature space based on the following decision function: $f\left(x\right)=sign\left(\sum_{i=1}^{l}\alpha_{i}^{0}y_{i}K(x,x_{i})+b\right)$, where the coefficients α_*i*_^*0*^ and *b* are determined by maximizing the following Langrangian expression: $\sum_{i=1}^{l}\alpha_{i}-\frac{1}{2}\sum_{i=1}^{l}\sum_{j=1}^{l}\alpha_{i}\alpha_{j}y_{i}y_{j}K\left(x_{i},x_{j}\right)$ under the conditions *α*_*i*_ ≥ 0 and $\sum_{i=1}^{l}\alpha_{i}y_{i}=0$. A positive or negative *f(****x****)* value indicates that the vector ***x*** belongs to the active or inactive class respectively.

In developing our SVM VS tool, a hard margin c=100,000 was used. The margin parameter c is penalty parameter that controls the trade-off between the training errors and sample separation. Increasing c imposes a higher penalty for training errors. Our chosen value corresponds to a very high penalty. The performance of SVM was evaluated by 5-fold cross-validation test. Table 2 shows the results of the 5-fold cross validation of SVM VS models of Src inhibitors and putative non-inhibitors. After the 5-fold cross-validation, the σ values were found to be 1.2 based on the average VS performance for the model development. Its performance indicators can be derived from the numbers of true positives *TP* (true inhibitors), true negatives *TN* (true non-inhibitors), false positives *FP* (false inhibitors), and false negatives *FN* (false non-inhibitors). Src inhibitor and non-inhibitor prediction accuracies are given by sensitivity *SE*=*TP/(TP+FN*)*100 and specificity *SP*=TN/(TN+FP)*100 respectively. Prediction accuracies have also been frequently measured by overall prediction accuracy (*Q*) and Matthews correlation coefficient (*C*)[67]

(3) $$Q=\frac{TP+TN}{TP+TN+FP+FN}$$

(4) $$C=\frac{TP*TN-FN*FP}{\sqrt{\left(TP+FN\right)\left(TP+FP\right)\left(TN+FN\right)\left(TN+FP\right)}}$$

In the large database screening tests, the yield and false-hit rate are given by *TP/(TP+FN)* and *FP/(TP+FP*) respectively.

**Table 2:** Performance of SVM for identifying Src inhibitors and non-inhibitors evaluated by 5-fold cross validation study

**Cross - validation**	**Src inhibitors**				**Src non-inhibitors**				**Q**	**C**
	**No of training/ testing inhibitors**	**TP**	**FN**	**SE**	**No of training/testing non-inhibitors**	**TN**	**FP**	**SP**		
1	1362/341	320	21	93.84%	50654/12664	12651	13	99.90%	99.74%	0.948
2	1362/341	324	17	95.01%	50654/12664	12650	14	99.89%	99.76%	0.953
3	1362/341	324	17	95.01%	50654/12664	12640	24	99.81%	99.68%	0.939
4	1363/340	318	22	93.53%	50655/12663	12642	21	99.83%	99.67%	0.935
5	1363/340	322	18	94.71%	50655/12663	12643	20	99.84%	99.71%	0.943
Average				94.42%				99.85%	99.71%	0.944
SD				0.0069				0.0004	0.0004	0.0072
SE				0.0031				0.0002	0.0002	0.0032

### Tanimoto similarity searching method

Compounds similar to at least one known Src inhibitor in a training dataset can be identified by using the Tanimoto coefficient *sim(i,j)*[68]

(5) $$sim\left(i,j\right)=\frac{\sum_{d=1}^{l}x_{\mathrm{di}}x_{\mathrm{dj}}}{\sum_{d=1}^{l}{\left(x_{\mathrm{di}}\right)}^{2}+\sum_{d=1}^{l}{\left(x_{\mathrm{dj}}\right)}^{2}-\sum_{d=1}^{l}x_{\mathrm{di}}x_{\mathrm{dj}}}$$

where *l* is the number of molecular descriptors. A compound *i* is considered to be similar to a known active j in the active dataset if the corresponding *sim(i,j)* value is greater than a cut-off value. In this work, the similarity search was conducted for MDDR compounds. Therefore, in computing *sim(i,j)*, the molecular descriptor vectors **x**_*i*_s were scaled with respect to all of the MDDR compounds. The cut-off values for similarity compounds are typically in the range of 0.8 to 0.9 [21,69]. A stricter cut-off value of 0.9 was used in this study.

### K-nearest neighbour method

kNN measures the Euclidean distance $D=\sqrt{{\left\|x-x_{i}\right\|}^{2}}$ between a compound ***x*** and each individual inhibitor or non-inhibitor ***x***_*i*_ in the training set[70]. A total of *k* number of vectors nearest to the vector ***x*** are used to determine the decision function *f(****x****)*:

(6) $$\hat{f}\left(x\right)\leftarrow \arg\max_{v\in V}\sum_{i=1}^{k}\delta \left(v,f\left(x_{i}\right)\right)$$

Where *δ(a,b)*=1 if *a=b* and *δ(a,b)=*0 if *a≠b*, argmax is the maximum of the function, V is a finite set of vectors {v1,…,vs} and $\hat{f}\left(x\right)$ is an estimate of f(x). Here estimate refers to the class of the majority compound group (i.e. inhibitors or non-inhibitors) of the k nearest neighbours. The performance of kNN was evaluated by 5-fold cross-validation in the same manner as in SVM and Table 3 shows the results of the 5-fold cross-validation results of kNN model. After the 5-fold cross-validation, the parameter k=1 was found to give the best performance of this work.

**Table 3:** Performance of kNN for identifying Src inhibitors and non-inhibitors evaluated by 5-fold cross validation study

**Cross - validation**	**Src inhibitors**				**Src non-inhibitors**				**Q**	**C**
	**No of training/ testing inhibitors**	**TP**	**FN**	**SE**	**No of training/testing non-inhibitors**	**TN**	**FP**	**SP**		
1	1362/341	302	39	88.56%	50654/12664	12635	29	99.77%	99.48%	0.896
2	1362/341	313	28	91.79%	50654/12664	12620	44	99.65%	99.45%	0.894
3	1362/341	311	30	91.20%	50654/12664	12610	54	99.57%	99.35%	0.878
4	1363/340	316	24	92.94%	50655/12663	12619	44	99.65%	99.48%	0.901
5	1363/340	302	38	88.82%	50655/12663	12632	31	99.76%	99.47%	0.895
Average				90.66%				99.68%	99.44%	0.893
SD				0.0191				0.0008	0.0005	0.0085
SE				0.0085				0.0004	0.0002	0.0038

### Probabilistic neural network method

PNN is a form of neural network that classifies objects based on Bayes’ optimal decision rule[71]*h*_*i*_*c*_*i*_*f*_*i*_(**x**) > *h*_*j*_*c*_*j*_*f*_*j*_(**x**), where *h*_*i*_ and *h*_*j*_ are the prior probabilities, *c*_*i*_ and *c*_*j*_ are the costs of misclassification and *f*_*i*_*(x)* and *f*_*j*_*(x)* are the probability density function for class *i* and *j* respectively. A compound ***x*** is classified into class *i* if the product of all the three terms is greater for class *i* than for any other class *j* (not equal to *i)*. In most applications, the prior probabilities and costs of misclassifications are treated as being equal. The probability density function for each class for a univariate case can be estimated by using the Parzen’s nonparametric estimator [72].

(7) $$g\left(x\right)=\frac{1}{n\sigma }\sum_{i=1}^{n}W\left(\frac{x-x_{i}}{\sigma }\right)$$

where *n* is the sample size, σ is a scaling parameter which defines the width of the bell curve that surrounds each sample point, *W(d)* is a weight function which has its largest value at *d* = 0 and (***x*** – ***x***_*i*_*)* is the distance between the unknown vector and a vector in the training set. The Parzen’s nonparametric estimator was later expanded by Cacoullos [73] for the multivariate case.

(8) $$g\left(x_{1},\ldots ,x_{p}\right)=\frac{1}{n\sigma_{1}\ldots \sigma_{p}}\sum_{i=1}^{n}W(\frac{x_{1}-x_{1,i}}{\sigma_{1}},\ldots ,\frac{x_{p}-x_{p,i}}{\sigma_{p}})$$

The Gaussian function is frequently used as the weight function because it is well behaved, easily calculated and satisfies the conditions required by Parzen’s estimator. Thus the probability density function for the multivariate case becomes

(9) $$g\left(x\right)=\frac{1}{n}\sum_{i=1}^{n}\exp(-\sum_{j=1}^{p}{\left(\frac{x_{j}-x_{\mathrm{ij}}}{\sigma_{j}}\right)}^{2})$$

The network architectures of PNN are determined by the number of compounds and descriptors in the training set. There are 4 layers in a PNN. The input layer provides input values to all neurons in the pattern layer and has as many neurons as the number of descriptors in the training set. The number of pattern neurons is determined by the total number of compounds in the training set. Each pattern neuron computes a distance measure between the input and the training case represented by that neuron and then subjects the distance measure to the Parzen’s nonparameteric estimator. The summation layer has a neuron for each class and the neurons sum all the pattern neurons’ output corresponding to members of that summation neuron’s class to obtain the estimated probability density function for that class. The single neuron in the output layer then estimates the class of the unknown compound ***x*** by comparing all the probability density function from the summation neurons and choosing the class with the highest probability density function. The performance of PNN was validated by 5-fold cross-validation in the same manner as in SVM model development. Table 4 shows the results of the 5-fold cross-validation of PNN model. After the 5-fold cross-validation, the parameter of the developed PNN models was chosen as 0.02.

**Table 4:** Performance of PNN for identifying Src inhibitors and non-inhibitors evaluated by 5-fold cross validation study

**Cross - validation**	**Src inhibitors**				**Src non-inhibitors**				**Q**	**C**
	**No of training/ testing inhibitors**	**TP**	**FN**	**SE**	**No of training/testing non-inhibitors**	**TN**	**FP**	**SP**		
1	1362/341	319	22	93.55%	50654/12664	12413	251	98.02%	97.90%	0.715
2	1362/341	324	17	95.01%	50654/12664	12380	284	97.76%	97.69%	0.702
3	1362/341	330	11	96.77%	50654/12664	12395	269	97.88%	97.85%	0.722
4	1363/340	330	10	97.06%	50655/12663	12389	274	97.84%	97.82%	0.720
5	1363/340	318	22	93.53%	50655/12663	12413	250	98.03%	97.91%	0.715
Average				95.19%				97.90%	97.83%	0.715
SD				0.0169				0.0012	0.0009	0.0075
SE				0.0076				0.0005	0.0004	0.0034

## Results and discussion

### Performance of SVM, kNN and PNN identification of Src inhibitors based on 5-fold cross validation test

**Figure 3:**
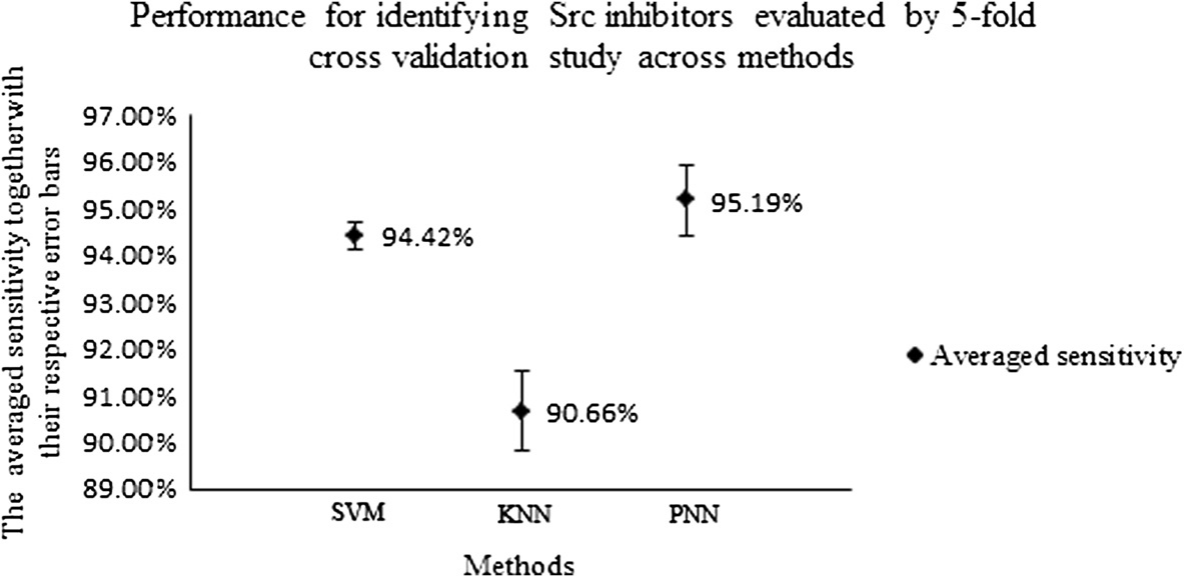
**Performance for identifying Src inhibitors evaluated by 5-fold cross validation study across methods.** Figure 3 is illustrating the 5-fold cross-validation studies of Src inhibitors across methods with the averaged sensitivity together with their respective error bars.

The parameters of our SVM, kNN and PNN models were determined by 5-fold cross-validation studies of Src inhibitors and non-inhibitors. The results of these tests for SVM, kNN and PNN are shown in Tables 2,3,4 and Figure 3 respectively. Overall, the sensitivity of SVM, kNN and PNN is in the range of 93.53%~95.01%, 88.56%~92.94% and 93.53%~97.06%, the specificity in the range of 99.81%~99.90%, 99.57%~99.77% and 97.76%~98.03%, and overall accuracy Q in the range of 99.67%~99.76%, 99.35%~99.48% and 97.69%~97.91% respectively. The inhibitor accuracies of our SVM are comparable to or slightly better than the reported accuracies of 58.3%~67.3% for protein kinase C inhibitors by SVM-RBF and CKD methods [74], 83% for Lck inhibitors by SVM method [75], and 74%~87% for inhibitors of any of the 8 kinases (3 Ser/Thr and 5 Tyr kinases) by SVM, ANN, GA/kNN, and RP methods [76]. The non-inhibitor accuracies are comparable to the value of 99.9% for Lck inhibitors [75] and substantially better than the typical values of 77%~96% of other studies [74,76]. Caution needs to be exercised about straightforward comparison of these results, which might be misleading because the outcome of VS strongly depends on the datasets and molecular descriptors used. Based on these rough comparisons, SVM appears to show good capability in identifying Src inhibitors at low false-hit rates.

### Virtual screening performance of SVM in searching Src inhibitors from large compound libraries

As outlined in the methods section, we developed a SVM VS tool for searching Src inhibitors from large were developed by using Src kinases reported before 2011. The VS performance of SVM in identifying Src inhibitors reported since 2011 and in searching MDDR and PubChem databases is summarised in Table 5. The yield in searching Src inhibitors reported since 2011 is 70.45%, which is comparable to the reported 50%~94% yields of various VS tools [77]. Strictly speaking, direct comparison of the reported performances of these VS tools is inappropriate because of the differences in the type, composition and diversity of compounds screened, and in the molecular descriptors, VS tools and their parameters used. The comparison cannot go beyond the statistics of accuracies.

**Table 5:** Virtual screening performance of support vector machines for identifying Src inhibitors from large compound libraries

**Inhibitors in training set**	**Number of inhibitors**	**1703**
	**Number of chemical families covered by inhibitors**	**493**
Inhibitors in Testing Set	Number of Inhibitors	44
	Number of Chemical Families Covered by Inhibitors	35
	Percent of Inhibitors in Chemical Families Covered by Inhibitors in Training Set	51.43%
Virtual Screening Performance	Yield	70.45%
	Number and Percent of Identified True Inhibitors Outside Training Chemical Families	15 (34.1%)
	Number and Percent of 13.56M PubChemCompounds Identified as Inhibitors	44,843 (0.33%)
	Number and Percent of the 168K MDDR Compounds Identified as Inhibitors	1,496 (0.89%)
	Number and Percent of the 9,305 MDDR Compounds Similar to the Known Inhibitors Identified as Inhibitors	719 (7.73%)

We also evaluated virtual-hit rates and false-hit rates of SVM in screening compounds that resemble the structural and physicochemical properties of the known Src inhibitors by using 9,305 MDDR compounds similar to an Src inhibitor in the training dataset. Similarity was defined by Tanimoto similarity coefficient ≥0.9 between a MDDR compound and its closest inhibitor [18]. This stricter similarity metric was used for conducting a stricter test of our SVM model. SVM identified 719 virtual-hits from these 9,305 MDDR similarity compounds (virtual-hit rate 7.73%), which suggests that SVM has some level of capability in distinguishing Src inhibitors from non-inhibitor similarity compounds. Significantly lower virtual-hit rates and thus false-hit rates were found in screening large libraries of 168 K MDDR and 13.56 M PubChem compounds. The numbers of virtual-hits and virtual-hit rates in screening 168 K MDDR compounds are 1,496 and 0.89% respectively. The numbers of virtual-hits and virtual-hit rates in screening 13.56 M PubChem compounds are 44,843 and 0.33% respectively.

Substantial percentages of the MDDR virtual-hits belong to the classes of antineoplastic, tyrosine-specific protein kinase inhibitors, signal transduction inhibitors, antiangiogenic, and antiarthritic (Table 6, details in next section). As some of these virtual-hits may be true Src inhibitors, the false-hit rate of our SVM is at most equal to and likely less than the virtual-hit rate. Hence the false-hit rate is <7.73% in screening 9,305 MDDR similarity compounds, <0.89% in screening 168 K MDDR compounds, and <0.33% in screening 13.56 M PubChem compounds, which are comparable and in some cases better than the reported false-hit rates of 0.0054%~8.3% of SVM [18,78], 0.08%~3% of structure-based methods, 0.1%~5% by other machine learning methods, 0.16%~8.2% by clustering methods, and 1.15%~26% by pharmacophore models [77].

**Table 6:** MDDR classes that contain higher percentage (≥3%) of SVM virtual-hits and the percentage values

**MDDR Classes that contain higher percentage (≥3%) of virtual hits**	**No of virtual hits in class**	**Percentage of class members selected as virtual hits**
Antineoplastic	623	2.9%
Tyrosine-Specific Protein Kinase Inhibitor	231	19.6%
Signal Transduction Inhibitor	194	9.5%
Antiarthritic	176	1.5%
Antiallergic/Antiasthmatic	83	0.8%
Antihypertensive	76	0.7%
Antiangiogenic	75	4.6%
Treatment for Osteoporosis	55	2.2%
Antidepressant	49	0.8%

Virtual-hits are identified by SVMs in screening 168K MDDR compounds for Src inhibitors. The total number of SVM identified virtual hits is 1,496.

### Experimental test of a SVM identified virtual-hit

**Figure 4:**
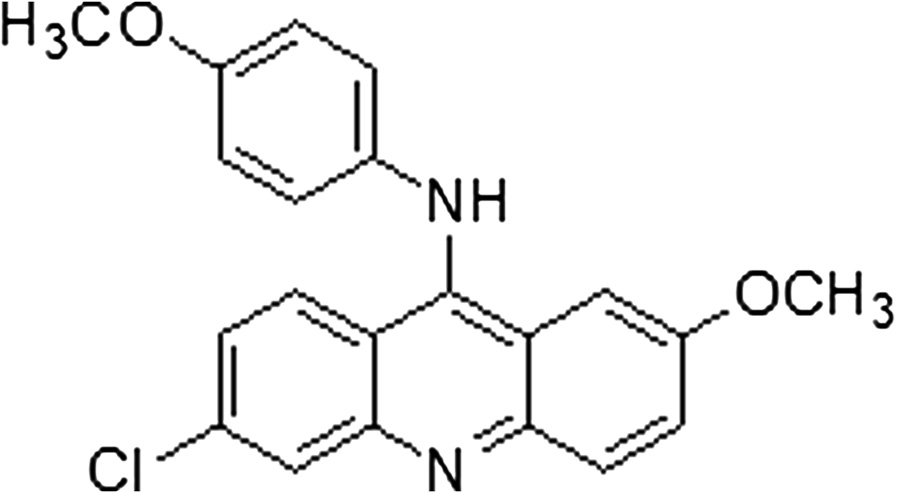
Virtual hit inhibiting Src at a moderate rate of 4.85% at 20 μM.

Three virtual hits of the same novel scaffold from in-house libraries not found in the known the Src inhibitor were evaluated for inhibitory activity against Src. Src kinase was incubated with substrates, compounds and ATP in a final buffer of 25 mM HEPES (pH 7.4), 10 mM MgCl_2_, 0.01% Triton X-100, 100 μg/mL BSA, 2.5 mM DTT in 384-well plate with the total volume of 10 μl. The assay plate was incubated at 30°C for 1h and stopped with the addition of equal volume of kinase glo plus reagent. The luminescence was read at envision. The signal was correlated with the amount of ATP present in the reaction and was inversely correlated with the kinase activity. One of three virtual hits showing in Figure 4 was found to inhibit Src at a moderate rate of 4.85% at 20 μM.

### Evaluation of SVM identified MDDR virtual-hits

SVM identified MDDR virtual-hits were evaluated based on the known biological or therapeutic target classes specified in MDDR. Table 6 gives the MDDR classes that contain higher percentage (≥3%) of SVM virtual-hits and the percentage values. We found that 623 (41.6%) of the 1,496 virtual-hits belong to the antineoplastic class, which represent 2.9% of the 21,557 MDDR compounds in the class. In particular, 231 (15.4%) of the virtual-hits belong to the tyrosine-specific protein kinase inhibitor class, which represent 19.6% of the 1,181 MDDR compounds in the class. Moreover, 194 (13.0%) and 75 (5.0%) of the virtual-hits belong to the signal transduction inhibitor and antiangiogenic classes, representing 9.5% and 4.6% of the 2,037 and 1,629 members in these classes respectively. Therefore, many of the SVM virtual-hits are antineoplastic compounds that inhibit tyrosine kinases and possibly other kinases involved in signal transduction and angiogensis pathways. While some of these kinase inhibitors might be true Src inhibitors, a significant percentage of them are expected to arise from false selection of inhibitors of other kinases.

A total of 176 (11.8%) SVM virtual-hits belong to the antiarthritic class. A primary feature of rheumatoid arthritis in synovial tissues is the abnormal stimulation of fibrin deposition, angiogenesis and proinflammatory processes, which are promoted by thrombin increased IL-6 production via the PAR1 receptor/PI-PLC/PKC alpha/c-Src/NF-kappaB and p300 signaling pathways [79]. Therefore, Src inhibitors may have some effects against arthritis via interference with some of these processes. Moreover, several other kinases have been implicated in arthritis. An Abl inhibitor Gleevec has been reported to be effective in treatment of arthritis, which is probably due to its inhibition of other related kinases such as c-kit and PDGFR [80]. EGFR-like receptor stimulates synovial cells and its elevated activities may be involved in the pathogenesis of rheumatoid arthritis [78]. VEGF has been related to such autoimmune diseases as systemic lupus erythematosus, rheumatoid arthritis, and multiple sclerosis [81]. FGFR may partly mediates osteoarthritis [82]. PDGF-like factors stimulates the proliferative and invasive phenotype of rheumatoid arthritis synovial connective tissue cells [83]. Lck inhibition leads to immunosuppression and has been explored for the treatment of rheumatoid arthritis and asthma [84]. Therefore, some of the SVM virtual-hits in the antiarthritic class may be inhibitors of these kinases or their kinase-likes capable of producing antiarthritic activities.

Moreover, 83 (5.5%), 76 (5.1%), 55 (3.7%) and 49 (3.3%) of the SVM virtual hits are in the antiallergic/antiasthmatic, antihypertensive, osteoporosis treatment and antidepressant classes respectively. Src or Src family kinases have been implicated in and the respective inhibitors have shown observable effects against these diseases. For instance, Src family kinases and lipid mediators have been found to partly control allergic inflammation [85]. Inhibition of Src family kinase-dependent signaling cascades in mast cells may exert anti-allergic activity [86]. Up-regulation of Src signaling has been suggested to be important in the profibrotic and proinflammatory actions of aldosterone in a genetic model of hypertension, which can be significantly reduced by mineralocorticoid receptor blocker and Src inhibitor [87]. Src signalling pathways play critical roles in osteoclasts and osteoblasts, and Src inhibitors have been developed as therapeutic agents for bone diseases [88,89]. Src-family protein tyrosine kinases negatively regulate cerebellar long-term depression, which can be recovered by the application of Src-family protein tyrosine kinase inhibitors [90]. Therefore, some of the SVM virtual hits in these four MDDR classes may be Src inhibitors or Src family kinase inhibitors capable of regulating allergic inflammation, hypertension, osteoporosis and depression respectively.

### Comparison of virtual screening performance of SVM with those of other vrtual screening methods

To evaluate the level of performance of SVM and whether the performance is due to the SVM classification models or to the molecular descriptors used, SVM results were compared with those of three other VS methods based on the same molecular descriptors, training dataset of Src inhibitors reported before 2011, and the testing dataset of Src inhibitors reported since 2011 and 168K MDDR compounds. The three other VS methods include two similarity-based methods, Tanimoto-based similarity searching and kNN methods, and an alternative machine learning method PNN. As shown in Table 7, the yield and maximum possible false-hit rate of the Tanimoto-based similarity searching, kNN and PNN methods are 36.84% and 5.54%, 38.64% and 2.49%, and 50.00% and 2.60% respectively. Compared to these results, the yield of SVM is better than these similarity-based VS method, and the false-hit rate of SVM is significantly reduced by 6.22, 2.80, and 2.92 fold respectively. These suggests that SVM performance is due primarily to the SVM classification models rather than the molecular descriptors used, and SVM is capable of achieving comparable yield at substantially reduced false-hit rate as compared to both similarity-based approach and alternative machine learning method. Our results are consistent with the report that SVM shows mostly good performances both on classification and regression tasks, but other classification and regression methods proved to be very competitive [19].

**Table 7:** Comparison of virtual screening performance of SVM with those of other methods

**Method**	**Inhibitors in training set**		**Inhibitors in testing set**			**Virtual screening performance**			
	**No of inhibitors**	**No of chemical families covered by inhibitors**	**No of inhibitors**	**No of chemical families covered by inhibitors**	**Percent of inhibitors in chemical families covered by inhibitors in training set**	**Yield**	**No and Percent of identified true inhibitors outside training chemical families**	**No and Percent of the 168K MDDR compounds identified as inhibitors**	**No and Percent of the 9,305 MDDR compounds similar to the known inhibitors identified as virtual inhibitors**
Support Vector Machines	1703	493	44	35	51.43%	70.45%	15(34.1%)	1,496 (0.89%)	719 (7.73%)
Tanimoto Similarity						36.84%	9(20.5%)	9,305 (5.54%)	9,305 (100%)
K Nearest Neighbour						38.64%	10(22.7%)	4,182 (2.49%)	1,169 (12.57%)
Probabilistic Neural Network						50.0%	13(29.5%)	4,386 (2.60%)	1,184 (12.72%)

### Does SVM select Src inhibitors or membership of compound families?

To further evaluate whether SVM identifies Src inhibitors rather than membership of certain compound families, compound family distribution of the identified Src inhibitors and non-inhibitors were analyzed. 34.1% of the identified inhibitors belong to the families that contain no known Src inhibitors. For those families that contain at least one known Src inhibitor, >70% of the compounds (>90% in majority cases) in each of these families were predicted as non-inhibitor by SVM. These results suggest that SVM identify Src inhibitors rather than membership to certain compound families. Some of the identified inhibitors not in the family of known inhibitors may serve as potential “novel” Src inhibitors. Therefore, as in the case shown by earlier studies [13], SVM has certain capacity for identifying novel active compounds from sparse as well as regular-sized active datasets.

## Conclusions

Our study suggested that SVM is capable of identifying Src inhibitors at comparable yield and in many cases substantially lower false-hit rate than those of typical VS tools reported in the literatures. It can be used for searching large compound libraries at sizes comparable to the 13.56 M PubChem and 168 K MDDR compounds at low false-hit rates. The performance of SVM is substantially improved against several other VS method based on the same datasets and molecular descriptors, suggesting that the VS performance of SVM is primarily due to SVM classification models rather than the molecular descriptors used. Three SVM virtual hits of the same novel scaffold were experimentally tested, one of which showed moderate Src inhibition rate. Because of its high computing speed and generalization capability for covering highly diverse spectrum compounds, SVM can be potentially explored to develop useful VS tools to complement other VS methods or to be used as part of integrated VS tools in facilitating the discovery of Src inhibitors and other active compounds [91-93].

## Competing interests

The authors declare that they have no competing interests.

## Authors’ contributions

BC Han conceived study, implemented the methods and wrote the manuscript with assistance from XH Ma, RY Zhao, JX Zhang, XN Wei, XH Liu, X Liu and YZ Chen. The experiments were conducted by CL Zhang, CY Tan and YY Jiang. All co-authors participated in study's design, coordination and manuscript drafting. All authors read and approved the final manuscript.
